# The Use of Quaternary Ammonium to Combat Dental Caries

**DOI:** 10.3390/ma8063532

**Published:** 2015-06-17

**Authors:** Yang Ge, Suping Wang, Xuedong Zhou, Haohao Wang, Hockin H. K. Xu, Lei Cheng

**Affiliations:** 1 State Key Laboratory of Oral Diseases, Sichuan University, Chengdu 610000, China; E-Mails: 2013224030068@stu.scu.edu.cn (Y.G.); 2012224030075@stu.scu.edu.cn (S.W.); zhouxd@scu.edu.cn (X.Z.); wanghaohao.aries@stu.scu.edu.cn (H.W.); 2 Department of Operative Dentistry and Endodontics, West China Hospital of Stomatology, Sichuan University, Chengdu 610000, China; 3 Biomaterials & Tissue Engineering Division, Department of Endodontics, Prosthodontics and Operative Dentistry, University of Maryland Dental School, Baltimore, MD 21201, USA; E-Mail: hxu@umaryland.edu

**Keywords:** quaternary ammonium monomer, dental caries, anti-biofilms, dental materials

## Abstract

Resin composites and adhesives are increasingly popular in dental restorations, but secondary caries is one of the main reasons for restoration failure. Quaternary ammonium monomers (QAMs) have an anti-microbial effect and are widely used in many fields. Since the concept of the immobilized antibacterial effect was put forward, dental restorations containing QAMs have been studied to reduce secondary caries. Previous studies have been struggling to develop novel anti-caries materials which might have triple benefits: good mechanical properties, antibacterial effects and remineralization potentials. Different kinds of QAMs have been proven to be effective in inhibiting the growth and metabolism of biofilms. Combination of QAMs and other nanoparticles in resin composites and adhesives could enhance their anti-caries capability. Therefore, QAMs are promising to show significant impact on the future of restorative and preventive dentistry.

## 1. Introduction

Dental caries, one of the most common bacterial infectious diseases in humans, is the localized destruction of susceptible dental hard tissues by acid produced from bacterial fermentation of dietary carbohydrates [1]. Dental caries is the primary cause of oral pain and tooth loss of human beings [2]. Resin composites and adhesive systems are increasingly popular for tooth cavity restorations because of their esthetics and direct-filling capability [3,4,5]. It was reported that 200 million dental restorations are placed annually in the U.S. [6]. Progresses have led to esthetic composite restoratives with less removal of tooth structures, enhanced load-bearing properties, and improved clinical performance [7,8,9,10,11,12,13]. However, previous *in vitro* studies indicated that resin composites accumulated more dental plaque than other restorative materials [14]. Secondary caries at the restoration margins is identified as a main limitation to the longevity of the restorations [15,16,17]. The replacement of existing restorations accounts for 50%–70% of all restorations [18,19]. Replacement dentistry costs more than $5 billion annually in the U.S. alone [20].

Investigators tried to synthesize ideal anti-caries dental materials which should have triple benefits: good mechanical properties, anti-biofilm effects and remineralization potentials. Traditional method for preparing antibacterial dental materials is to endow them with low-molecular-weight antibacterial agents, such as antibiotics, silver ions, chlorhexidine, and fluoride, which released gradually over time [21,22]. The low-molecular-weight antimicrobial agents have the shortcoming of the residual toxicity of the agents endangering the environment, and their effects are short lived because of the difficulty of controlling their rate of diffusion. Another disadvantage of dental materials that release antimicrobial agents is an adverse influence on mechanical properties [23].

**Figure 1:**
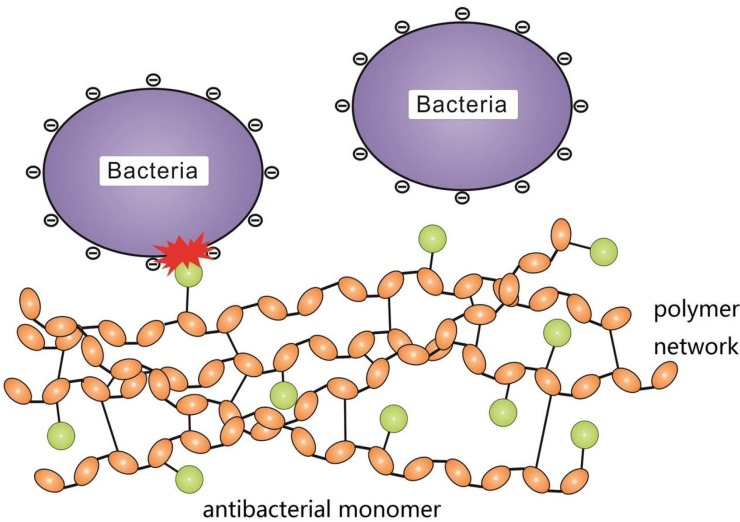
Schematic illustration of the antibacterial mechanism of quaternary ammonium monomers.

Quaternary ammonium salts (QAS) are widely used in water treatment, food industry, textiles and surface coating because of their low toxicity and a broad spectrum of antimicrobial activity [24]. The antibacterial mechanism of QAS is due to theircapability of causing bacteria lysis by binding to bacterial membranes [25,26,27]. When the negatively charged bacteria cells contact the positive quaternary amine charge (N^+^), the electric balance is disturbed and the bacterium could explode under its own osmotic pressure [25,26,27]. Long cationic polymers can penetrate bacterial cells to disrupt membranes, like needle bursting balloons [28,29] (Figure 1). There are numerous studies on synthesis of novel quaternary ammonium monomers [30], in order to find a compound which has several benefits, including good antibacterial effect, low cytotoxicity, without compromising mechanical properties, low cost and convenience of receipt. Antibacterial quaternary ammonium monomers have been incorporated into composite materials to inhibit plaque accumulation and secondary caries since nearly 30 years ago. In the 1970s, QAS were first incorporated into mouth rinses to inhibit oral biofilms [31,32]. In order to achieve long-term antibacterial effectiveness without compromising in mechanical properties, a concept of “immobilized bactericide” was introduced into dentistry [33,34]. Imazato *et al.* alsofirst incorporated a quaternary ammonium monomer into dental composite materials in 1994 [35]. Since then, different kinds of QAMs (Table 1), including 12-methacryloyloxydodecylpyridinium bromide (MDPB) and methacryloxylethylcetylammonium chloride (DMAE-CB), quaternary ammonium dimethacrylate (QADM), quaternary ammonium polyethylenimine (QPEI) and so on, have been synthesized and incorporated into composites, such as glass ionomer cement (GIC), etching-bonding systems, and resin composites to achieve antibacterial effect. The review summarized the previous studies of dental materials incorporated with QAM. 

**Table 1:** Chemical structures of different quaternary ammonium monomers.

Quaternary Ammonium Monomers		Chemical Structure
Momomethacrylate quaternary ammonium	MDPB	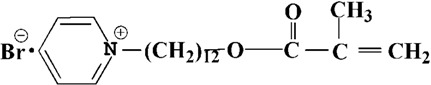
	DMAE-CB	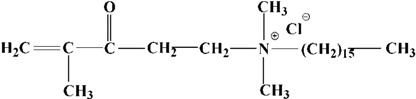
Dimethacrylate quaternary ammonium	QADM	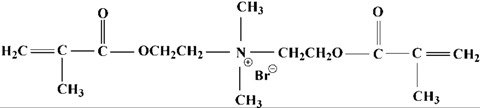
QPEI		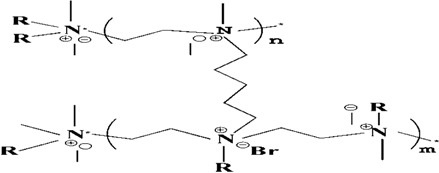

## 2. Monomethacrylate Quaternary Ammonium

### 2.1. MDPB

MDPB, a compound of quaternary ammonium dodecylpyridinium bromide and a methacryIoyl group, is synthesized according to a two-step route, first is to achieve methacryloyloxydodecylpyridinium bromide (MDB), and then purified MDB isconverted to MDPB by reaction with pyridine and purified. MDPB is the first quaternary ammonium monomer as an immobilized antibacterial agent incorporated into a commercially available self-etching adhesive systemwhich contains 5% MDPB [36].

The antibacterial effect of MDPB has been widely studied in previous published papers [33,36,37]. Un-polymerized MDPB acts as a free bactericide and has extremely strong antibacterial effect on various oral bacteria according to minimum inhibitory concentration (MIC) and minimum bactericidal concentration (MBC) values. MIC of MDPB against seven oral streptococci was reported, including *Streptococcus mutans*, *Streptococcus sobrinus*, *Streptococcus oralis*, *Streptococcus mitis*, *Streptococcus* *sanguis*, *Streptococcus gordonii*, *Streptococcus salicarius*, which ranged from 7.8 μg/mL to 25 μg/mL [35]. The MBC of MDPB against the seven oral streptococci also ranged from 31.3 μg/mL to 62.5 μg/mL [38]. In another study, strong antibacterial activity of MDPB against anaerobes was reported, with the MIC and MBC values ranging from 3.9~31.3 μg/mL and 15.6~125 μg/mL, respectively [39]. MDPB has a similar inhibitory activity against the growth of both bacteria related to dental caries and plaque formation. An *in vitro* study demonstrated that the killing effect of MDPB on *S. mutans* was proven by a sensitive means, viability staining tests, and the conventional plating method [37]. In order to investigate the antibacterial effect on organisms associated with active root caries lesions, clinical isolates from the root caries lesion was used to measure MICs and MBCs [40]. The taxa represented the dominant members of the microflora of root caries, including *Streptococcus mutans*, *Streptococcus oralis*, *Streptococcus salivarius*, *Actinomycesgerensceriae*, *Actinomycesodontolyticus*, *Lactobacillus* spp. and *Candida albicans* [41,42]. The median MICs and MBCs of MDPB against a range of organisms isolated from active root carious lesions were in the range of 3.13 to 25.0 μg/mL and 6.25 to 50.0 μg/mL, respectively [40] (Table 2).

MDPB monomer has a significant antibacterial effect on oral microflora associated with dental caries, and thennovel antibacterial dental materials incorporating MDPB are synthesized to combat caries. An *in vitro* study reported that cured MDPB-containing primer showed an inhibitory effect on the growth of all the bactreria which were in contact with the specimens’ surface, and displayed a little bactericidal effect on *S. mutans* without releasing any unpolymerized antibacterial components [43]. Another study showed that an experimental adhesive resin was prepared by incorporation of 2.5% MDPB into proprietary adhesive. The cured experimental adhesive exhibited an inhibitory effect on *S. mutans* growth, reducing the number of bacteria to approximately 3% of control adhesive without MDPB [44]. Composite incorporating of MDPB had a long-lasting antibacterial effect according to Imazato *et al.*’s study. No elution of the antibacterial components was observed from the material, even after 90 days of immersion in water or other solvents. And *S. mutans* accumulated to a lesser degree on the surface of composite incorporating MDPB [35]. MDPB showed significant bacteriostatic effects without releasing antibacterial componentsand was useful for incorporation into various resin-based restoratives [33]. The antibacterial effects of the dentin primer, Clearfil Protect Bond, containing 5% MDPB were also demonstrated by *Imazato et al.* [36]. The capability to disinfect cavities containing residual bacteria, were tested by counting the viable bacteria of the impregnated dentin after application of the MDPB-containing primer to bacteria-impregnated dentin, which showed no formation of bacterial colony, meaning the bacterial recovery at <10 CFU (Colony-Forming Units) [36].

**Table 2:** MIC and MBC values of MDPB against different bacteria species.

Bacteria	MIC (μg/mL)	MBC (μg/mL)
*Streptococcus mutans* NCTC10449	15.6	62.5
*Streptococcus sobrinus* SL-1	7.8	62.5
*Streptococcus oralis* NCTC7864	16.7	31.3
*Streptococcus mitis* NCTC10712	25	31.3
*Streptococcus sanguis* NCTC7863	16.7	31.3
*Streptococcus gordonii* NCTC7868	16.7	31.3
*Streptococcus salicarius* NCTC8618	15.6	31.3
*Propionibacterium acnes* ATCC6919	3.9	62.5
*Eubacterium alactolyticum* ATCC23263	31.3	125
*Bifidobacterium bifidum* ATCC29521	31.3	62.5
*Peptostreptococcus asaccharolyticus* ATCC14963	31.3	31.3
*Lactobacillus plantarum* ATCC14917	7.8	15.6
*Lactobacillus salivarius* ssp. *Salivarius* ATCC11741	7.8	62.5
*Lactobacillus acidophilus* ATCC4356	15.6	62.5
*Lactobacillus paracasei* spp. *paracasei* ATCC27216	15.6	62.5
*Lactobacillus brevis* JCM1059	15.6	31.3
*Lactobacillus salivalius* ssp. *Salicinius* ATCC11742	15.6	125
*Lactobacillus fermentum* ATCC14931	15.6	15.6

In order to enhance the antibacterial effect of MDPB, dual agents like nanoparticles of silver (NAg) and MDPB were also incorporated into dental adhesive by Zhang *et al.* [45,46]. One study showed primer containing 5% MDPB and 0.05% NAg had a much stronger reduction in biofilm viability and acid production than MDPB or NAg alone, increasing inhibition zone size and reducing metabolic activity, CFU and lactic acid by an order of magnitude [46]. The other study demonstrated that the greatest antibacterial effect was achieved by adding 2.5% MDPB and 0.1% NAg into both adhesive and primer [45,46]. 

The influence on the mechanical properties of Bis-GMA-based composites, after MDPB incorporated into, has been investigated. A study by Imazato *et al.* studied the curing behavior of MDPB containing composites. The results demonstrated that depth of cure of composites with MDPB, was greater than a control material without MDPB. Additionally, the light-attenuating effect of MDPB composites was less than for the control. After both one day and seven days of storage in water, with respect to Vickers hardness, no significant difference between resin composite with MDPB and thecontrol group was found [47]. Bond strength to human dentin and degree of conversion of the experimental adhesive in combination with 1%, 2%, or 5% MDPB-containing primers were evaluated by conventional tensile bond strength test and Fourier transformation infrared spectroscopy. Tensile bond strength of experimental adhesive was not significantly different from that of the control, and combination with MDPB-containing primer did not show any adverse influence on bond strength at experimental concentrations [44]. A study using an animal model, via SEM examination and microtensile bond strengths test, confirmed that the experimental antibacterial adhesive systems employing MDPB-containing primer or/and bonding-resin could produce an effective bond under *in vivo* conditions [48]. Another study showed that using an adhesive system containing antibacterial monomer-MDPB, Clearfil Protect Bond, increased the shear bond strength of restorations luted with three dual-polymerizing systems [49].

Cytotoxic effect of MDPB is one of the important concerns for its clinical potential. It wasreported that no cytotoxic effect was observed on contact with MDPB at concentrations of 10 μg/mL or less [38]. MDPB showed cytotoxicity on human pulpal cells at a concentration above 20 μg/mL, and the 50% toxicity (ID_50_) of MDPB for human pulp cells lies between 20–40 μg/mL [38]. However, Ratanasathien *et al.* investigated the cytotoxicity of the momomers routinely used for dentin bongding systems against mouse fibrolasts [50]. The ID_50_ at 24 h contact had been reported to be 468 μg/mL for HEMA, 4.79 μg/mL for Bis-GMA, 35.7 for TEGDMA, and 8.68 μg/mL for UDMA. Imazato *et al.* suggested that MDPB was not more cytotoxic than the monomers used for resin-based dental materials, though there might be a little different between animal cells and human cells [38]. Even after 1 h of contact with the cured experimental primer, no elution of unpolymerized MDPB was detected [43]. Another study investigated the cytotoxic effects of self-etching primers containing MDPB to the human pulp cells. Incorporation of MDPB into a proprietary primer up to 5% had no significant influence on the cytotoxicity observed [51]. Zhang *et al.* studied the dual agents, MDPB and NAg, adding to adhesive system with experimental concentration, without showing cytotoxity against human fibroblast cells [45]. In addition, Ma *et al.* researched about *N*-acetyl cysteine(NAC) potentially reducing the toxicity of MDPB, and adduct formation being partially responsible for the detoxification ability of NAC against MDPB-induced cell damage[52].

### 2.2. DMAE-CB

Three novel experimental QAS monomers that are structurally different from the above monomers are developed, including methacryloxylethyl benzyl dimethyl ammonium chloride (DMAE-BC), methacryloxylethyl m-chloro benzyl dimethyl ammonium chloride (DMAE-m-CBC) and methacryloxylethylcetyl ammonium chloride (DMAE-CB). The MICs and MBCs against *Streptococcus mutans*, *Actinomycesvisosus*, *Lactobacuilluscase*i and *Staphylococcus aureus* were measured. DMAE-CB showed the best antibacterial activity, with MIC values of 1.2~4.8 μg/mL against the four strains [53]. Thereby DMAE-CB had been chosen to do further studies.

The antibacterial effect of a resin-based adhesive containing DMAE-CB against *S.mutans* was evaluated. DMAE-CB was incorporated in Single Bond 2 (3M ESPE, Minnesota, USA) at 1%, 2% and 3% (*w/w*) [54]. Antibacterial property after 0, 30, 90, and 180 days of aging was also tested. The cured experimental adhesive exhibited an inhibitory effect on *S. mutans* growth, and the effect increased with the larger concentration incorporated. Antibacterial activities of the specimens lasted for at least 180 days [54]. Another study investigated the effects of the cured adhesives and their eluents on the growth of *S. mutans* were determined by film contact test and absorbance measurement, respectively [55]. The effects of the cured adhesives on the adherence and membrane integrity of *S. mutans* were investigated using confocal laser scanning microscopy (CLSM) in conjunction with fluorescent indicators [55]. The results showed no detectable antibacterial activity of the eluents. Moreover, the fluorescence analysis of CLSM images demonstrated that the cured DMAE-CB-incorporated adhesive and positive control could inhibit the adherence of *S. mutans* and exert detrimental effect on bacterial membrane integrity [55]. The influence of DMAE-CB incorporated adhesive on the biofilm formation and *gtf* gene expression of *Streptococcus mutans*. The results showed significant decrease in biofilm accumulation on its surface, and suppression of the expression of *gtf*B and *gtf*C of *Streptococcus mutans* in the biofilm [56]. Bonding ability of the experimental adhesive incorporating 3% DMAE-CB was evaluated by microtensile bond strength test, but the results showed no adverse effects [54].

## 3. Dimethacrylate Quaternary Ammonium

### 3.1. QADM

MostQAMs incorporated into dental materials aremonomethacrylate, which generally have only one methacrylate group. Incorporating high concentrations of monomethacrylate could significantly affect the overall polymer network structures and mechanical properties. In addition, some monomethacrylates with pendant quaternary ammonium moieties may present miscibility problems with hydrophobic dimethacrylates commonly used in dental composites [57]. In a recent study, Antonucci *et al.* adapted the Menschutkin reaction for the synthesis of free radical, photo-crosslinking, dimethacrylate monomers containing quaternary ammonium functionalities [57]. One of the ionic dimethacrylate monomers (IDMAs) was referred as quaternary ammonium dimethacrylate (QADM) in other published papers [58,59,60].

There are several advantages of QADM. Firstly, the synthetic method for QADM was fairly straightforward when compared with the synthesis of other QAMs. A modified Menschutkin reaction was used andthe synthesis method is desirable because the reaction products are generated at quantitative amounts and required no further purification [57].

Secondly, compared with other QAS monomethacrylates, QADM has reactive groups on both ends of the molecule, which could be incorporated into the resin with less of a negative impact on the mechanical and physical properties of the composite. Thus, QAMs have been added into different kinds of dental materials containing resin matrix, including resin composite and adhesive systems, without compressing the mechanical properties [61]. In an *in vitro* experiment, different mass fractions of QADM were incorporated into resin composites. The results indicated that even with 18% QADM, the composite strength still matched that of the two commercial composites without antibacterial properties. Increasing the QADM mass fraction produced a monotonic increase in the antibacterial potency of the resin composite [58]. When QADM was added into a commercial or an experimental primer and adhesives, the mass fraction of this monomer could be as high as 10%, and the dentin shear bond strength of adhesives didn’t decrease compared with the control group [60]. However, when the amount of QADM was added to a certain limit, the mechanical properties would compromise. So the amount of QADM added into dental materials to study the antibacterial effects is up to the results of mechanical studies [58,59,60].

Thirdly, QADM was proven to be anti-bacterial/anti-biofilm and the effects were lasting. In previous studies, QADM has been added into resin composites and adhesives. Most of the investigations were *in vitro* experiments using *S mutans* biofilms and microcosm biofilms [58,59,61,62,63]. Resin composites containing 18% QADM had biofilm CFU, metabolic activity, and acid production abouthalf of those without QADM. Besides, inversely linear relationshipswere established between QADM mass fraction and *S. mutans* biofilm CFU, metabolic activity, and acid production [58]. In order to test whether the antibacterial capability and mechanical properties of QADM nanocomposite would decrease with water-aging time, a novel nanocomposite containing QADM was immersed into distilled water for six months, and its mechanical and antibacterial durability were studied via *in vitro* experiments. The results demonstrated that the antibacterial properties of NACP+QADM were maintained even after water-aging for 30, 90, and 180 days [59].

Incorporation of QADM into dental materials has several advantages in preventing dental caries, but investigators still tried to modify dental materials containing QADM to enhance the anti-caries and mechanical properties of those novel materials. There are three groups of micro-particles or nanoparticles added into dental materials along with QADM. The first group of particles is incorporated to promote the mechanical strength of resin composite or adhesives. In several papers, the mechanical properties of QADM resin composites were significantly better due to glass particle reinforcement [21,22,58,62]. According to Xu *et al.*’s study, a barium boroaluminosilicate glass of a mean particle size of 1.4 Lm (Caulk/Dentsply, Milford, DE, USA) was silanized with 4% 3-methacryloxypropyltrimethoxysilaneand 2% n-propylamine [64]. The second group is composed of other antibacterial agents, like nano-silver. The combination of two kinds of antibacterial nanoparticles could have stronger inhibiting effects on dental biofilms [22,62]. An *in vitro* study formulated antibacterial primers by incorporating QADM and NAg, and investigated their effects on plaque microcosm biofilms [60]. The results indicated that primers containing QADM and NAg had two benefits: (1) uncured primer could kill the planktonic bacteria in caries cavity. Another study was designed to support the antibacterial effect of primer containing with QADM and Nag [65]; (2) When the primer containing QADM and NAg was cured with adhesives, it could inhibit the dental biofilms. Zhang *et al.* developed an antibacterial bonding agent by incorporation of QADM and nanoparticles of silver and investigated the effects of QADM-NAg adhesive and primer on dentin bond strength and plaque microcosm biofilm response [66]. The third group refers to the calcium compounds as a portion of the filler phase for remineralization purposes [67,68,69,70]. Calcium phosphates, tetracalcium phosphate have been added into the QADM composite. These composites released supersaturating levels of calcium (Ca) and phosphate (PO_4_) ions and remineralized tooth lesions *in vitro*. Recently, novel CaP nanoparticles of sizes of about 100 nm were synthesized viaa spray-drying technique and filled into dentalcomposites. These nanocomposites achieved Ca andPO_4_ release similar to those of previous CaP composites, whilepossessing much better mechanical properties. In other papers, an experimental primer and adhesives were synthesized by incorporating nanoparticles of silver, QADM, and nanoparticles of amorphous calcium phosphate (NACP). The anti-caries effects were tested by applying microcosm biofilm model [71,72].

### 3.2. QPEI

QPEI nanoparticles, was synthesized by Beyth *et al.*, which was made from cross-linked polyethyleneimine (PEI) that was *N*-alkylated with octylhalide, followed by quaternary methylation with methyl iodide [26,73]. In order to study the antibacterial effect of QPEI-containing dental composite resins cured by light polymerization, three clinically used bonding, flowable and hybrid composite resin, embedding at 1% w/w QPEI, was studied in an *in vitro* investigation. The antibacterial activity was tested with *Streptococcus mutans* by the agar diffusion test, the direct contact test, and bacterial growth in the materials elute and scanning electron microscope (SEM). The results indicated that quaternary ammonium PEI nanoparticles immobilized in resin-based materials have a strong antibacterial activity upon contact without leach-out of the nanoparticles [26]. Shvero *et al.* [74] incorporated QPEI into a provisional cement at 0.5% w/w, 1% w/w and 2%w/w, and investigated the antibacterial effect against *Strepcococcus* *mutans* and *Enterococcus faecalis* using direct contact test. A strong antibacterial effect was evident for 1% w/w QPEI-containing cement after an aging period of 14 days against both *S. mutans* and *E. faecalis* [74]. Another study investigated the antibacterial effect of QPEI nanoparticles incorporated at 1% or 2% w/w in a resin composite [75]. The antimicrobial effect against *Staphylococcus* *aureus*, *Staphylococcus epidermidis*, *Enterococcus faecalis*, *Pseudomonas aeruginosa* and *Escherichia coli* was tested using the direct contact test (DCT), agar diffusion test (ADT) and scanning electron microscopy (SEM) [75]. 2% QPEI-containing composite resins inhibited the growth of the above bacteria strains. Reducing the amount of the added nanoparticles to 1% w/w resulted in complete inhibition of *S. aureus* and *E. faecalis*, and decreased growth of *S. epidermidis*, *P. aeruginosa* and *E. coli*. The DCT results were confirmed by SEM. ADT showed no inhibition halo in all test bacteria, indicating the antimicrobial nanoparticles are not diffusing into the agar milieu [75]. Another study adding 1% w/w QPEI into Filtek Flow showed that the antibacterial effect could last at least three months [76]. The studies of QPEI added to root canal sealer showed antibacterial effects against anaerobes and facultative anaerobes [77,78,79,80].

The mechanical properties of QPEI-containing composites are also important for the future applications, but only few studies mentioned them. A study showed the flexural modulus and the flexural strength of 1% w/w QPEI-containing composites resins had insignificant difference with the commercial ones [26]. The cytotoxicity of the QPEI-containing composite resin wasalso investigated both *in vitro* and *in vivo*. In the *in vitro* study, the cytotoxity effect on MBT cell lines was estimated by the [^3^H]-thymidine incorporation method [76]. The modified and unmodified dental composite resin showed a similar effect on the viability of cell lines [76]. *In vivo* cytotoxity studies, which were assessed on Wistar rats by the implantation of modified composite specimens, revealed no inflammation response 1 week after the implantation of restorative composite resin that was embedded with up to 2% *w/w* QPEI [76]. Then, biocompatibility tests via macrophage viability and TNFa secretion revealed that those were not altered by the presence of QPEI nanoparticles in the resin [75]. Recently, a study *in vivo* suggested that resin composites containing 1% wt/wt QPEI exerted a significant antibiofilm activity and exhibited a potent broad spectrum antibacterial activity against salivary bacteria [81]. Generally, QPEI nanoparticles are a promising antibacterial monomer in the future clinical application, but further studies are needed to investigate the mechanical properties.

## 4. QAS with Different Chain Lengths

Increasing the alkyl chain length (CL) increased the hydrophobicity, which could enhance the propensity to penetrate the hydrophobic bacterial membrane. Therefore, cationic polymers with longer CL could be more effective in penetrating bacterial cells to disrupt membranes [28,29]. Therefore, some investigators tried to synthesize QAS with different chain lengths and then studied their anti-caries potentials in different dental materials. A recent study on glass ionomers showed that increasing the chain length significantly increased the antibacterial potency [82]. In another study, a series of QAS compounds with different chain lengths were synthesized, including dimethylami-nopropyl methacrylate (DMAPM, CL=3), dimethylamino-hexyl methacrylate(DMAHM, CL=6), dimethylaminononyl methacrylate (DMANM, CL=9), dimethylaminododecylmethacrylate (DMADDM, CL=12), dimethylaminohexadecyl methacrylate(DMAHDM, CL=16) and dimethylaminooctadecyl methacrylate (DMAODM, CL=18) [83] (Table 3). Then, two of those antibacterial monomers were selected for investigation, including DMADDM with a chain length of 12 and DMAHM with a chain length of six. DMAHM and DMADDM were much more strongly antibacterial than the previous QADM in MIC, MBC and ADT tests. Furthermore, DMADDM with a chain length of 12 was far more potent than DMAHM with a chain length of six [63]. Among those monomers, DMADDM with CL=12 and DMAHDM with CL=16 were widely studied as anti-caries components in different kinds of dental materials because of their strong antibacterial effects. 

**Table 3:** Quaternary ammonium monomers of different chain length.

Monomer	Chain Length	Chemical Structure
DMAPM	3 (*n* = 2)	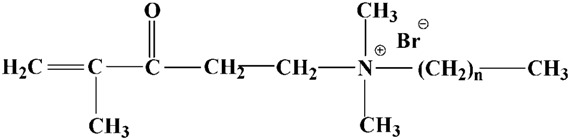
DMAHM	6 (*n* = 5)	
DMANM	9 (*n* = 8)	
DMADDM	12 (*n* = 11)	
DMAHDM	16 (*n* = 15)	
DMAODM	18 (*n* = 17)	

DMADDM were added into resin composite, adhesives and orthodontic cement as anti-caries agents [63,84,85]. Since restorations *in vivo* are exposed to saliva, one concern is the attenuation of antibacterial activity due to salivary pellicles. An *in vitro* experiment was designed to investigate the effects of salivary pellicles on bonding agents containing DMADDM or NAg against microcosm biofilms. The results indicated that novel DMADDM- and NAg-containing bonding agents substantially reduced biofilm growth even with salivary pellicle coating on surfaces, indicating a promising usage in saliva-rich environment[86].Another study by Wang *et al.* was to incorporate different mass fractions of DMADDM into commercial adhesives and to investigate the inhibiting effects on *S. mutans* biofilms and dentin bonding properties. It indicated that lower mass fractions of DMADDM had similar antibacterial effects as higher mass fractions of DMADDM on initial biofilms. However, adhesives with 5% DMADDM showed stronger anti-biofilm potential on the mature biofilm than the adhesives with 2.5% DMADDM. Further, the dentin adhesive bond strength of the novel antibacterial adhesives in the present study matched that of the control commercial product [87].The antibacterial effect of DMADDM-containing adhesive on multispecies biofilms, formed by *Streptococcus mutans*, *Streptococcus gordonii*, and *Streptococcus sanguinis*, was studied recently by Zhang *et al.* [88]. In addition, the proportion change in multispecies biofilms with different mass fractions of DMADDM was also studied by Taqman real-time polymerase chain reaction. The results showed that the ratio of *Streptococcus mutans* in biofilms increased in the adhesives without DMADDM, and that of *Streptococcus gordonii* decreased continuously over time. However, in the adhesives containing DMADDM, the ration of *Streptococcus mutans* dropped noticeably and the ratio of *Streptococcus gordonii* was increasing steadily [88]. *Streptococcus gordonii* is reported as an early colonizer of the dental plaque biofilm and is associated with sound enamel [89]. Thus, the biofilm has a more healthy development tendency after the regulation of DMADDM.

Another advantage of DMADDM-containing adhesives is to inhibit matrix metalloproteinases (MMPs) and enhance the dentin–resin bonding durability. The degradation of the hybrid layer at the dentin–adhesive interface was believed to be the primary reason for failure, which was caused by several mechanical and chemical factors, including the hydrolysis and enzymatic degradation of the exposed collagen and the adhesive resin [90]. Recently, it was reported that host-derived MMPs were involved in hybridlayer degradation [91]. Li *et al.* investigated the effects of DMADDM on soluble rhMMP-8 and rhMMP-9 and human dentin matrix-bond endogenous MMPs, and on dentin elastic modulus and dentin dissolution and mass loss. The results indicated that DMADDM had potent inhibitory effects against soluble rhMMP-8 and rhMMP-9 and matrix-bond endogenous MMPs; the use of DMADDM greatly reduced the elastic modulus loss in demineralized dentin, dentin mass loss, and the dissolution of collagen peptides from dentin, compared to control without DMADDM [92].

Though DMADDM was demonstrated to have much stronger antibacterial effect on the dental plaque, several studies still tried to combine DMADDM with other anti-caries agents in different dental materials. Chen *et al.* developed bonding agent with the double benefits of antibacterial and remineralizing capabilities by adding DMADDM and nanoparticles of amorphous calcium phosphate (NACP) into primer and adhesive [93]. Incorporating DMADDM into bonding agent yielded a potent antibacterial activity, while NACP in adhesive released Ca and P ions for remineralization and caries-inhibition. This was achieved without compromising the dentin bond strength at one day and one month. In another long-term experiment, bonding agents containing DMADDM and NACP were water-aged for six months, and the results indicated that the novel anti-caries adhesives yielded potent and long-lasting antibacterial properties and much stronger bond strength after six months of water-ageing than a commercial control [94].

The CL effects of QAS on cytotoxicity were also investigated in another *in vitro* experiment. QAS with chain length of three to 16 had fibroblast/odontoblast cytotoxicity similar to those of commercial controls [95]. The cytotoxicity of DMADDM on human gingival fibroblasts (HGF) was assessed using a methyl thiazolyltetrazolium assay and live/dead viability assay. The results indicated that DMADDM had much lower cytotoxicity than BisGMA, which is widely used in commercial products [96]. Further studies were carried out to investigate restoratives containing NACP and DMADDM in a rat tooth model, and examine pulpal inflammation and tertiary dentin formation. It demonstrated that adding DMADDM had little effect on pulpal inflammation, compared with using a commercial adhesive and a glass-filled composite [97].

DMAHDM with chain length of 16 were also added into resin composites and adhesives to synthesize novel antibacterial dental materials. Adhesives containing 10% DMAHDM could reduce microcosm biofilm CFU by 4 log [83]. In another *in vitro* study, DMAHDM was mixed into adhesives and primers at mass fractions of 0%, 2.5%, 5%, 7.5%, and 10%. Bacteria early-attachment coverage greatly decreased with increasing DMAHDM mass fraction in the resin. However, incorporation of DMAHDM didn’t compromise the dentin bond strength [98]. In order to get double or triple benefits in preventing dental caries, DMAHDM were added into dental caries combining with other effective agents, like NACP, 2-methacryloyloxyethyl phosphorylcholine (MPC) and so on [99,100].

## 5. Conclusions

QAM is a promising antibacterial monomer. QAMs containing dental materials can perform a long-lasting antibacterial effect on bacteria associated with dental caries, without compromising mechanical properties, due to the property of QAMs combining with resin matrix. Now, no safety problems have been reported, but no studies last more than 12 months. QAMs, strong antibacterial agents, like other bactericides, may induce drug resistance. More studies need to be done about the biocompatibility, especially *in vivo*.
